# Phenomenological Studies of Visual Mental Imagery: A Review and Synthesis of Historical Datasets

**DOI:** 10.3390/vision7040067

**Published:** 2023-10-20

**Authors:** David F. Marks

**Affiliations:** Independent Researcher, 13200 Arles, Bouches-du-Rhône, Provence-Alpes-Côte d’Azur, France; dfmarksphd@gmail.com

**Keywords:** phenomenology, visual mental imagery, after-imagery, eidetic imagery, memory imagery, vividness, clarity, colourfulness, liveliness, projection, metamorphosis, individual differences

## Abstract

This article reviews historically significant phenomenological studies of visual mental imagery (VMI), starting with Fechner in 1860 and continuing to the present. This synthesis of diverse VMI phenomenological studies in healthy adults serves as a unique resource for investigators of individual differences, cognitive development and clinical and neurological conditions. The review focuses on two kinds of VMI, “memory imagery” and “eidetic imagery”. Ten primary studies are drawn from three periods of the scholarly literature: early (1860–1929), middle (1930–1999) and recent (2000–2023). It is concluded that memory and eidetic imagery are two forms of constructive imagery, varying along a continuum of intensity or vividness. Vividness is a combination of clarity, colourfulness and liveliness, where clarity is defined by brightness and sharpness, colourfulness by image saturation and liveliness by vivacity, animation, feeling, solidity, projection and metamorphosis. The findings are integrated in a template that specifies, as a tree-like structure, the 16 properties of VMI vividness in healthy adult humans. The template takes into account the weight of evidence drawn from the accounts and reveals an extraordinary degree of consistency in reported VMI characteristics, revealed by specialized studies of healthy adult humans across time, space and culture.

## 1. Introduction

### 1.1. Purpose

Any stable environment of familiar objects evokes sensations, images and feelings that vary by degree across individuals. Accordingly, it is surprising that any pair of individuals is able to reach agreement about what they are perceiving and feeling based on the two sets of fallible conscious experiences. While everyone may have their own unique perspective, there must also exist rules that create consistency. Anyone can experience the “redness” of an apple, the “pointedness” of a stick or “the warm feeling” of a campfire because there is, one must believe, something that it is like to have these experiences. There are particular phenomenological qualities or “qualia” that each of us has with these experiences [1]. The redness, roundness and warmth that we experience share sufficient commonality and consistency to enable us to communicate about them. What appears curious about this surprising fact rests not only with the mysterious qualia themselves, but with the interesting fact that qualia are triggered by sensorial objects and also by absent objects that are available only as mental images. Three tantalizing questions remain to be answered: what is it like to experience mental imagery, what is mental imagery for, and how are we to explain the striking individual differences? Books and journals are filled with accounts of theories and experiments, but a cohesive description of the phenomenology of mental imagery appears lacking. In attempting to answer the above-mentioned questions, one needs to review the findings of phenomenological studies, which is the purpose of the present review.

Almost every reviewer of mental imagery research starts with Aristotle (384–322 B.C.E.), who stated that “Images belong to the rational soul in the manner of perceptions, and whenever it affirms or denies that something is good or bad, it pursues or avoids. Consequently, the soul never thinks without an image” [2]. Skipping forward 22 centuries, we are not surprised to discover a few complications and technical hitches including, for example, Maurice Merleau-Ponty’s assertion that “we can understand that nuances and sensation, perception and imagery are all ambiguous terms, even for what the psychologist of perception appears as an essence, e.g., figure vs. ground, can implode with the pressure of ambiguity” [3]. On occasion, philosophers appear confused about mental imagery, and one can discover perplexing claims such as the following: “It does not seem like mental imagery is an ordinary language term… and no languages other than English has (sic) a term that would mean mental imagery (as distinct from ‘imagination’ or ‘mental picture’)”. The encyclopaedia continues, “This encyclopaedia entry will not attempt to give an ordinary language analysis of the term “mental imagery”, partly because it is far from clear that ‘mental imagery’ is part of the ordinary language” [4]. These statements appear perplexing because, to the best of the author’s knowledge, the leading world languages manifestly do all have a term for “mental imagery”, e.g., Chinese: 心理意象 (929 M speakers), Spanish: “imágen mental” (475 M speakers), Hindi: मानसिक कल्पना (422 M speakers), Arabic: “الصورةالذهنية” (313 M speakers), French: “imagerie mentale” (300 M speakers), Russian: мыcлeнныe oбpaзы (258 M speakers) and German: “mentales Bild” (155 M speakers). Moreover, as is the case for English, these seven languages all use a different word for “imagination”. Thus, for billions of speakers, the term “mental imagery”—or its precise lexical equivalent—is a part of the ordinary language, and the term “mental imagery” is distinct from the term for “imagination”. I do not dwell any further here on philosophical views but move on to consider the foundations of our knowledge concerning the phenomenology of visual mental imagery (VMI).

**Figure 1:**
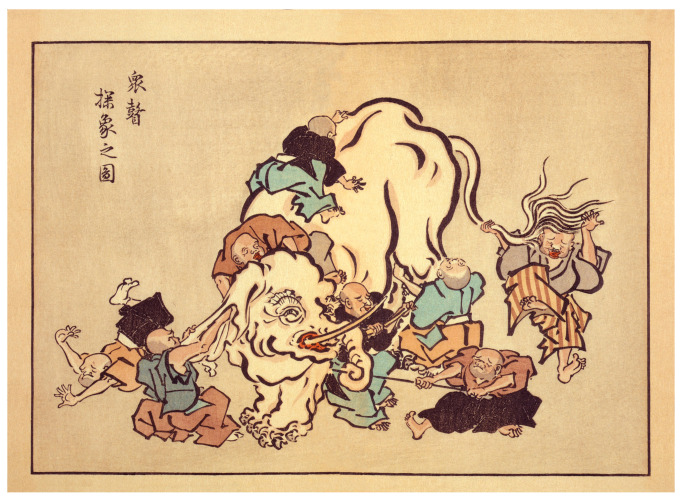
The parable of the blind monks inspecting the different parts of an elephant (Public Domain).

The study of visual mental imagery since the so-called “Cognitive Revival” has received the theoretical attention of multiple psychologists [5,6,7,8,9,10,11,12,13,14]. However, without any agreement, clarity or precision about the phenomenological nature of visual mental imagery experiences, mental imagery investigators are acting like the blind monks examining the different parts of an elephant in the well-known parable (Figure 1).

My purpose here is to synthesize a collection of individual studies of the “elephant” that is visual mental imagery in an integrative review of VMI phenomenology. This review encompasses studies utilizing a variety of procedures for imagery evocation, including introspective analyses by competent observers, imagery volunteered in response to questionnaires, perceptual presentations, special instructions, interviews, or spontaneous evocations while listening to, or reading, texts. The majority of included studies are not experiments—although some were called such—but they have generated a sufficiently detailed description of imagery content to enable principles and processes to be compared and contrasted across participants and studies.

The ability to compare and contrast people’s accounts of VMI is necessary if we are to progress our knowledge about the nature and purpose of VMI. Elsewhere, I have suggested that mental imagery is a foundation of conscious experience with six constituent modular processes: schemata, objects, actions, affect, goals and others’ behaviour [15]. The evidence supporting these six connected modules and their interconnections provides empirical support from a wide range of studies, including neuroscientific studies, which show that all six modules of perception and mental imagery are associated with wide individual differences in the production of vividness [15]. The goal here is to explore the nature of produced VMI through phenomenological studies.

Traditionally, researchers have divided VMI into four kinds: after-images, eidetic images, memory images and imagination images, e.g., [16]. As noted, this review focuses on two of these four kinds, eidetic imagery and memory imagery, although necessarily there is some cross-over and “blending” between these two kinds and the other two kinds. As we shall see, the qualities, content and characteristics of VMI can be benchmarked against the objects of perceptual imagery.

Mental imagery research is replete with disciplinary shifts of interest, theoretical debates and bouts of “literature blindness”, when the same phenomenon can be “discovered”, re-labelled and republished over and over again. In the mental imagery field, one could say that there has been a surplus of theoretical assumptions and laboratory experiments but a dearth of phenomenological studies, which, one can only surmise, has been detrimental to progress. To the best of this author’s knowledge, this is the first review of the psychological literature on VMI phenomenology covering the period from Fechner’s 1860 landmark work to the present day. It is helpful to define the terms used here in reference to VMI.

### 1.2. Glossary of Terms

After-image (or perceptual after-image): one of four main kinds of mental image, in which an image is retained after the cessation of a stimulus that is externally projected, according to the principles of Emmert’s Law.

Brightness (opp. dim): objects with the appearance of good illumination.

Clarity: a combination of brightness and sharpness, i.e., having a distinguishable outline and details.

Corporeity: the appearance of solidity, of having or being a body.

Easel Test: a procedure for testing the ability to produce eidetic images by presenting and then removing a picture on an easel for a fixed time.

Eidetic image: from the Greek “eidos”, meaning idea or something seen, a persistent, vivid, projected image known to be subjective, so not confused with real objects.

Emmert’s Law: as described by Emmert in 1881, retinal images that are perceived to differ in physical size when localized at different distances according to the formula: Perceived size = Retinal image size × Perceived distance.

Hallucination: a persistent, vivid, projected image that is mistakenly thought to be a real object.

Imagination image: a mental image of an object or combination of objects never previously experienced.

Individual differences: reliably observed differences between individuals in any mental, behavioural or physical attribute.

Introspective report: a description of mental experience within a particular set and setting.

Liveliness: a combination of vivacity, projection, solidity, animation, feeling and metamorphosis.

Memory image: a mental image of something previously experienced.

Mental image: a quasi-perceptual experience of an object or activity in the absence of the object or activity.

Metamorphosis: unplanned changes in image content that follow a sequence based on colour, form and meaning.

Multi-modal mental image: mental imagery that includes visual and/or auditory, olfactory, gustatory and tactile elements.

Open Circle Test: a procedure for testing a person’s ability to produce eidetic images while looking at a circle, combined with hearing a stimulus in the form of a colour name.

Phenomenological study: an investigation of a person’s experience of any form of mental activity.

Projection: the impression of an external spatial location and three-dimensionality.

Self-report: a person’s description of a mental experience.

Sharpness (opp. vague): the quality of having a distinct outline and details.

Vivacity: the quality of being animated, affect-laden and active.

Vividness: a combination of clarity, colourfulness and liveliness.

### 1.3. Methods and Ground Rules

Methods for the phenomenological investigation of VMI have varied according to ideological positions about the nature of psychology that have held favour at different times. Typically, data have consisted of introspective reports by research participants in response to inquiries, interviews, questionnaires and “think-aloud” techniques in experimental settings (see Glossary, Section 1.2 above). More rarely, non-verbal methods such as drawing, dance, play, games, music and performance have been utilized. Here, some use is made of drawings. However, for practical reasons, VMI in the context of artistic media could not be included in this review. The review also necessarily avoids the intrinsic difficulties of mental imagery studies with children. Although research has often favoured the use of children, their questionable introspective abilities and the potential for the biasing of accounts with demand characteristics can be significantly reduced by employing adult participants. This review is concerned with the VMI of healthy adults, and research by Binet [17], Charcot [18] and Piaget [19] on child development, psychopathological and neurological conditions is not included.

The purpose is to construct a synthesis in the form of a “template” of VMI in healthy adult humans. Odd though it may seem, given the intensive efforts of psychologists to investigate mental imagery, our core knowledge about the content and characteristics of VMI has yet to be fully documented. Until now, no synthesis of knowledge about the range of content and characteristics of VMI in the healthy adult population has been produced. Without a template for the normal range of individual VMI capabilities, characteristics and contents, specialists in child development, psychopathology or neurological conditions are, in one real sense, working “in the dark” because they lack a normative standard.

The approach is to review “classic” and contemporary studies that have evaluated the visual mental imagery experiences of individuals to standardized introspective interviews, procedures and questionnaires. To be included in this review, an investigation must have recorded the participants’ self-reported, i.e., verbal or non-verbal description, VMI content in response to a standard test, questionnaire, interview or other data-collection procedure. Random, anecdotal reflections do not meet the criteria. The datasets from the selected studies are quantitatively evaluated using objective criteria such as frequencies, averages and total scores for criteria that were defined by the original investigators themselves.

For example, Fechner ascertained his participants’ answers to questions regarding whether it was easier to form visual mental images with their eyes open or closed, whether there was vividness or colourfulness to some degree, were there any feelings of pressure, contraction or effort, and whether it was possible to project the image to an external location. Reading Fechner’s accounts, it is possible to categorize participants’ responses according to each criterion, to tabulate the data and to arrive at an overall profile of the sample’s distribution of responses. Galton focused his inquiry on illumination— is the image dim or fairly clear? Is its brightness comparable to that of the actual scene? Definition— are all the objects pretty well defined at the same time, or is the place of sharpest definition at any one moment more contracted than it is in a real scene? and colouration. Other criteria include the ability to project mental imagery into space, three-dimensionality, relative size, multi-sensorial attributes, movement, controllability, emotion or feeling and somatic aspects such as tension in or around the eyes.

A similar procedure must be applied to each and every dataset. Whenever possible, the same or similar criteria would be sought in different investigations, as would a distribution of participants’ responses that could be tabulated, compared and aggregated across studies. Using these procedures, it is possible to harmonize and integrate different datasets into a criterion-based template in the form of a hierarchical description of visual mental imagery phenomenology, based on the investigators’ and research participants’ language about mental imagery experiences.

**Figure 2:**
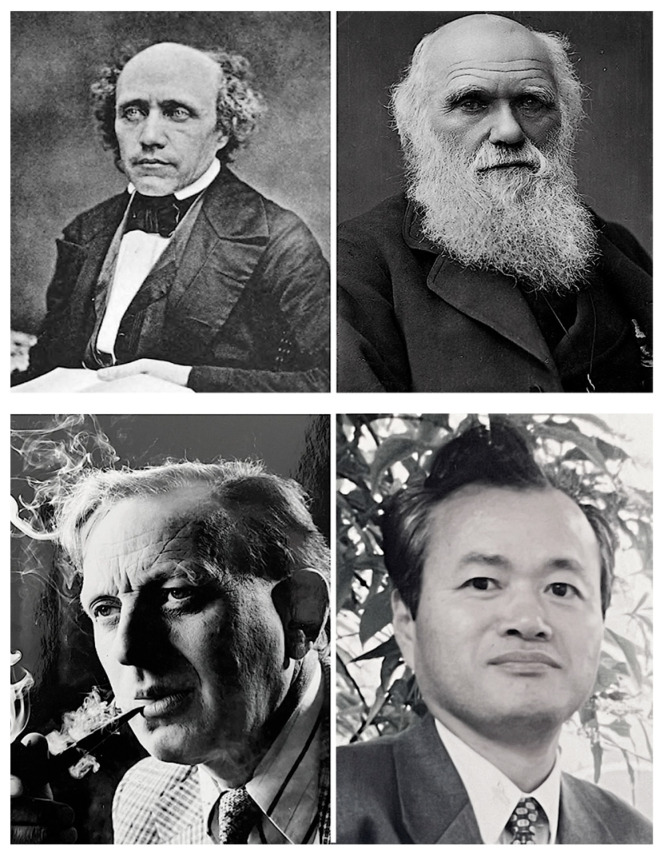
(**Top left**): Gustav Fechner, 1801–1887, the leading early investigator of VMI (Public domain); (**Top right**): Charles Darwin, 1809–1882, Galton’s half-cousin, and a participant in Galton’s studies included here (Public domain); (**Bottom left**): Peter McKellar, 1921–2003, a leading investigator from the middle period (photograph from the author’s collection); (**Bottom right**): Takeo Hatakeyama of Tohoku University, Japan, a leading investigator from the middle period (photograph from the author’s collection, © David F Marks).

The studies to be reviewed are drawn from three historical periods: early (1860–1929), middle (1930–1999) and recent (2000–2023), which are considered in turn. Three of the leading investigators, together with Charles Darwin, a participant in one of the early studies, are depicted in Figure 2.

## 2. The Early Period: 1860–1929

### 2.1. Gustav Fechner’s (1860) Observations on After-Images and Memory Images

The phenomenological study of mental images began in earnest with the renowned German scientist, Gustav Fechner [20]. As the founder of psychophysics and co-developer of the Weber–Fechner Law, Fechner presented the world with a unique treasure trove of phenomenological observations on VMI that has been sorely neglected by anglophones. In his two-volume work, *Elemente der Psychophysik* (1860), Fechner stated the following:

*“Initially, and in general, it cannot be denied that the mental sphere is subject to quantitative considerations. After all we can speak of a greater or lesser intensity of sensation; there are drives of different strengths, and greater and lesser degrees of attention, of the vividness of images of memory and fantasy, and of clearness of consciousness in general, as well as of the intensity of separate thoughts”.*

([20], p. 46)

Until now, only Volume I of Gustav Fechner’s (1860) *Elemente der Psychophysik* had been available in English [20]. Here, I review Fechner’s account of VMI in Volume 2 of *Elemente der Psychophysik*, chapter XLIV [21]. Fechner writes:

*“If the memory-images, fantasy-images and schemata accompanying thought are all still psychophysically founded, so is thought itself, in that every other substance and course of thought presupposes another material and another way of linking the schemata, without which no thought can take place at all, just as another melody and harmony cannot be without other tones and another way of linking the tones. Now a piano, with its comparatively small number of fixed keys, nevertheless affords the possibility of executing the most diverse melodies and harmonies, and however many and however high thoughts man may conceive, 25 letters suffice to express them; in both cases it depends only on the connection and the sequence in which the keys or letters are passed through. The brain, however, with its innumerable fibres, active in various ways, contains incomparably richer means in this respect, so there can be no obstacle to trusting it with at least as great a performance inwardly as we perform outwardly by means of it”*.

[20]

Fechner’s observations [21] concerning after-imagery, memory imagery and comparisons between them, were based on interviews with six professorial and professional colleagues, including his wife, and introspections of his own mental imagery. The recent translation of Fechner’s [21] mental imagery research is prefixed with Fechner’s remarks, as follows:

*Sensory impressions once made from the outside continue to exist for a certain time after the removal of the external stimulus as after-images, after-sounds, generally as after-perceptions, which in a healthy, strong state of the senses tend to be less easily perceived, less intense and lasting than in a weakly stimulable state; and they leave behind the capacity to be reproduced in memories or more or less transformed in phantasy images. Both kinds of after-effects are to be considered here mainly, if not exclusively, in the field of facial perception, where they have been most studied; but what is valid here is more or less applicable to other fields of sense perception.*

*The main differences between after-images on the one hand, and memory and phantasy images on the other, are that the former are only ever accompanied by a feeling of receptivity. The first always arise and exist only with a feeling of receptivity, only in continuity with the sensory impressions made, independent of volition and association with the imagination, and, according to the immediately preceding sensory impressions, also proceed independently of volition, legitimately, whereas the memory and phantasy images, with a feeling of lesser or greater spontaneity, can arise even a long time after preceding sensory influences, partly involuntarily through association with the imagination, partly volitionally, and can be banished and altered again.*

([21], pp. 3–4)

Thus, Fechner makes a distinction between the after-image as *involuntary*, and memory and imagination images as *volitional* or *partly volitional*, and so can be “extinguished” by an act of will or the needs of the moment. Fechner discusses his own imagery ability as follows:

*In general, memory and fantasy images always appear to me as something lacking in corporeality, airy, breathy, in contrast to the more material impression of the after-images.*

*Thus, the drawing of the memory and fantasy images is more vague and blurred than that of the after-images. I am not able to obtain clear, sharp outlines even on the most familiar memory images of the objects that are daily before my eyes, while the after-images appear with corresponding sharpness as directly seen objects.*

*After-images in the closed eye are either deeper black or lighter than the surrounding ground of the eye and the uniform black of the field of vision, depending on the brightness of the objects viewed in relation to the ground on which they appeared. Memory images, on the other hand, generally give me a weaker impression than the black itself. From white to black there is a scale of continuously graduated brightness and the deepest black is the pure black of the eye. If I now ask myself where this scale would lead if I were to think of it as continuing below black, I believe that one is led to the indistinct impression of memory and fantasy images.*

*With all my efforts, I cannot reproduce colours in the memory images of coloured objects, or only in fleeting, doubtful appearances when recalling very striking impressions (for example, when I think of cut eggs on spinach, where the white, yellow and green stand out very sharply against each other) while I receive vivid coloured after-images in the open as well as the closed eye. I also never dream in colours, but all my experiences in dreams seem to me to proceed in a kind of twilight or night.*

*I am not able to recall even the most familiar memory-images.*

*It is not possible to hold on to the image steadily, even for a short time, but in order to look at it longer, it must, so to speak, always be recreated anew; it does not both change of its own accord and disappear again and again of its own accord. If, however, I want to reproduce it often one after the other with the same intention, it soon no longer succeeds at all, for the attention or activity of production soon dulls. This, however, is not a dulling of memory activity in general; for I am not prevented—and this seems to me worthy of attention—from immediately imagining another familiar memory image instead, as clearly as it is at all possible for me to do so, and, when attention or production activity has also exhausted itself for this one, to return to the first image where I can produce it again with the initial clarity. This is true even of quite related pictures; as, for example, I have often attempted with two portrait figures on the same photograph or portraits hanging next to each other in my living room, neither of which I can often reproduce in memory one after the other, but both in repeated alternation. If, however, I continue this alternation somewhat quickly and often one after the other, I finally find myself dulled for both pictures, but can pass on to a third picture with success.*

*I cannot change after-images at all by will.*

([21], pp. 4–5)

For economy of space, I tabulate a summary of the remainder of Fechner’s observations in Table 1.

From the results of his “experiments”, Fechner noted the following:

*“(1) Sometime after the creation, the figures disappear or change into others without my being able to prevent this. (Fechner’s report of figures disappearing and changing into others without control matches the phenomena of metamorphosis described later in this paper).*

*(2) If the colour does not belong integrally to an object, I do not always have it completely under my control. A face, for example, never appears blue to me, but always in its natural colour, whereas instead of the imaginary red cloth, a blue one can appear at times; in general, the production of a certain colour is more difficult than that of a certain shape, and the first one succeeded in my p. 485*

*I did not succeed in the first attempts, since I had already succeeded in the last.*

*(3) I have succeeded a few times in seeing pure colours without objects; they then filled the entire field of vision.*

*(4) Objects that are not familiar to me, i.e., mere fantasy images, I often do not see, and instead of them, familiar objects of the same kind appear to me; for example, I once wanted to see a brass sword handle with a brass basket, but instead I saw the more familiar image of a rapping basket.*

*(5) Most of these subjective appearances, especially if they were bright, leave after-images if the eyes are opened quickly during the dwelling of the appearance; for example, I thought of a silver stirrup, and after looking at it for a while, I opened my eyes and saw the dark after-image of it for a long time.” (Fechner’s report of the after-imagery of memory imagery has been verified by later observers, e.g., [22,23])*.

[21]

In addition to seven cases, Fechner discusses accounts of mental imagery by figures such as Johann Wolfgang von Goethe, Gerolamo Cardano and others. These anecdotal observations presage findings reported by later investigators. For example, the first-person account attributed to Goethe, is golden:

*“Goethe says in Contributions to Morphology and Natural Science: “I have the gift, when I close my eyes and, with my head bowed down, think of a flower in the center of my visual organ, it does not remain for a moment in its first form, but it spreads out and from within it unfolds again new flowers of coloured, even green, leaves; they are not natural flowers, but fantastic, but regular, like the rosettes of the sculptors. It is impossible to foresee the sprouting creation, but it lasts as long as I like, does not tire and does not intensify. I can produce the same if I think of the ornament of a colourfully painted disk, which then also changes continuously from the center to the periphery, completely like the kaleidoscopes.”*.

([21], p. 18)

Goethe’s account of the spontaneous metamorphosis of VMI content is the first known description of the phenomenon studied by psychologists two centuries later (see Section 3).

Fechner next discusses the account of Gerolamo Cardano (1501–1576), the Italian polymath and mathematician, “who tells of himself that he had been able to imagine luminously what he wanted”. Then, Alexandre Brierre de Boismont (1797–1881), who discussed the mental imagery reported by an unidentified, prodigious portrait painter:

*“One painter, who had inherited a large part of the clientele of the famous Sir Joshua Reynolds, and believed him to be of superior talent to his own, was so busy that he confessed to me, says Wigan, that he had painted 300 portraits, large and small, in one year. This fact seems physically impossible; but the secret of his speed and astonishing success was this: he needed only one sitting to represent the model. I saw him execute before my eyes in less than eight hours the miniature portrait of a gentleman whom I knew very well; it was done with the greatest care and a perfect likeness.*

*I asked him to give me some details of his process, and this is what he replied: “When a model came along, I looked at it attentively for half an hour, sketching on the canvas from time to time. I didn’t need any longer. I would remove the canvas and move on to another person. When I wanted to continue the first portrait, I took the man in my mind, I put him on the chair, where I saw him as clearly as if he were ‘616’ written in the sky; and I can even add with sharper and more vivid shapes and colours. I looked from time to time at the imaginary figure, and I began to paint; I suspended my work to examine the pose, absolutely as if the original had been before me; every time I cast my eyes on the chair, I saw the man.”*

([21], pp. 18–19])

Fechner’s findings warrant the following conclusions about VMI:

(i) Wide individual differences were evident in both vividness and colouration;
(ii) Some people reported stronger imagery with their eyes open, others with their eyes closed and others reported imagery of equal strength with their eyes open or closed;
(iii) Fechner’s participants reported an impression of pressure, contraction or tension in generating VMI;
(iv) In all seven cases, VMI was projected into external space, corresponding to normal vision;
(v) In four cases, including Fechner himself, solid-looking, three-dimensional images could be formed;
(vi) In the same four cases, multi-sensory imagery spontaneously arose;
(vii) The cases Fechner presented comprised seven intellectuals, six men and one woman. As Fechner was aware, larger, more diverse samples would be required to form more definitive conclusions. The VMI characteristics in this study were evoked under Fechner’s particular instructions and may not generalize to other procedures for inducing VMI, e.g., spontaneous VMI while reading, listening or thinking.

### 2.2. Galton’s Breakfast Table Questionnaire

In 1880, a widely cited but misinterpreted study by Sir Francis Galton [24,25] reported findings obtained with his “Breakfast Table Questionnaire”, which Galton gave to 100 “men of science”, including his half-cousin, Charles Darwin, and numerous friends and colleagues. Galton also administered the same questionnaire to 172 schoolboys, whom he divided into two groups, “to serve as a check upon one another”. Group A comprised boys of the four upper classes in the school, group B those of the five lower classes. The first questions were presented as follows:

*“Before addressing yourself to any of the Questions on the opposite page, think of some definite object—suppose it is your breakfast-table as you sat down to it this morning—and consider carefully the picture that rises before your mind’s eye*.

(p. 302)

*1. Illumination.—Is the image dim or fairly clear? Is its brightness comparable to that of the actual scene?*

*2. Definition.—Are all the objects pretty well defined at the same time, or is the place of sharpest definition at any one moment more contracted than it is in a real scene?*

*3. Colouring.—Are the colours of the china, of the toast, bread-crust, mustard, meat, parsley, or whatever may have been on the table, quite distinct and natural?”*

Wide individual differences in the vividness, projection and corporeity of responses were obtained, which Galton divided into three categories: cases where the faculty is *very high*, mediocre or *at the lowest.* One curious feature of Galton’s own statements about his findings that has received critical comment was his claim to have been “amazed” at the poor or non-existent ability of the sample of scientists to mentally image. In fact, the results show the exact opposite. In only less than a handful of cases—perhaps only one out of one hundred—was zero imagery power reported. The vast majority of 99% reported at least some imagery ability. We return to this point later.

The first three of the “very high” category stated the following:

*1. Brilliant, distinct, never blotchy.*

*2. Quite comparable to the real object. I feel as though I was dazzled, e.g., when recalling the sun to my mental vision.*

*3. In some instances quite as bright as an actual scene.*

In the “mediocre” category, the first three people stated the following:

*46. Fairly clear and not incomparable in illumination with that of the real scene, especially when I first catch it. Apt to become fainter when more particularly attended to.*

*47. Fairly clear, not quite comparable to that of the actual scene. Some objects are more sharply defined than others, the more familiar objects coming more distinctly in my mind.*

*48. Fairly clear as a general image; details rather misty.*

The last three cases in the lowest category stated the following:

*98. No. My memory is not of the nature of a spontaneous vision, though I remember well where a word occurs in a page, how furniture looks in a room. The ideas are not felt to be mental pictures, but rather the symbols of facts.*

*99. Extremely dim. The impressions are in all respects so dim, vague and transient, that I doubt whether they can reasonably be called images. They are incomparably less than those of dreams.*

*100. My powers are zero. To my consciousness there is almost no association of memory with objective visual impressions. I recollect the breakfast table, but do not see it.*

To compare different samples along comparable scales, Galton [24] invented a system whereby each of his three samples were divided into octiles. A high degree of consistency was obtained across octiles from the three samples. Galton’s findings are summarized in Table 2.

Galton’s observations permit the following conclusions:

(i) Similar to Fechner’s data, Galton’s samples reported wide individual differences in the vividness and colourfulness of their VMI;
(ii) Also in line with Fechner’s data, some people reported stronger imagery with their eyes open, others with their eyes closed;
(iii) Consistent with Fechner’s findings, in a large majority of cases, mental images were localized in external space, corresponding to normal vision;
(iv) In the majority of cases, the apparent field of view in mental imagery was enlarged or the same as normal vision;
(v) In several cases, three-dimensional visual imagery could be formed, again confirming Fechner’s findings;
(vi) In at least some cases, multi-sensory images were formed.

Galton’s observations were limited to men of high socio-economic status, so they lacked diversity, limiting the generality of the conclusions to the human population as a whole. As noted above, Galton mis-stated his findings in alleging a lack of mental imagery ability in his sample of male scientists [24,25]. Galton’s “amazement” was plainly inconsistent with the facts. Galton’s observations did not in the very least support his claims about the deficient visual imagery of scientists. In a literature review, Brewer and Schommer-Atkins [26] found close to 50 secondary sources from 1883 to the present day that repeated, in parrot fashion, Galton’s conclusion that scientists were totally lacking in visual imagery or had only “feeble” powers of mental imagery. This episode indicates a curious tendency in academic psychology towards “literature blindness”, a kind of cognitive myopia, in which past errors are repeated without correction. Galton’s conclusion is contradicted by the responses of Charles Darwin, who described the definition of his VMI “as distinct as if I had photos before me” and “perfectly coloured” [26].

Brewer and Schommer-Atkins attempted to replicate Galton’s study with contemporary scientists and undergraduates and found *none* totally lacking in visual imagery, and only very few with feeble visual imagery. Brewer and Schommer-Atkins stated that Galton’s conclusions were a “theory-laden interpretation of data based on the initial responses from several very salient scientists who reported little or no visual imagery on Galton’s imagery questionnaire” ([26], p.130).

### 2.3. Armstrong’s Replication of Galton’s Studies

In the early 1890s, A. C. Armstrong [27] replicated Galton’s Breakfast Table study in the USA, with the assistance of C H Judd. The investigators gave Galton’s original questionnaire to 188 men, 37 at Princeton College (1881–1882) and 151 at Wesleyan University (1890–1893) [24]. According to their answers about “illumination”, Armstrong divided the sample into five classes: “This class-division based on 1. is in general one of the most important that can be made” ([27], p. 498). The answers to Galton’s question four—“What difference do you perceive between a very vivid mental picture called up in the dark and a real scene ?”—showed “a close connection between the degree of illumination and the sense of reality”. Retaining the same five-fold division for question two, Armstrong found two “natural” subdivisions under each class: “A, those subjects who have all the objects on the imaged table pretty well defined at the same time, and B, those whose place of sharpest definition is somewhat contracted”. The replies to question three indicated, again, a widespread individual difference in the visualization of colour, with 172 of 188 subjects (91.5%) thinking that their colour imagery was relatively “distinct and natural”, while only 16 (8.5%) failed in this respect although a few of these could “bring out the missing quality by turning their attention toward it”.

The answers to question five also showed remarkable consistency with those obtained by Galton: “Distance of Images.—Where do mental images appear to be situated—within the head, within the eyeball, just in front of the eyes, or at a distance corresponding to reality? Can you project an image upon a piece of paper?”. The majority of Armstrong’s students (133 or 71%) stated that the distance corresponded to reality, while six saw their images at positions more distant than the real scene, “as in a kind of bird’s eye view”. Of the remaining 49, 19 localized their images in the head, 14 just in front of the eyes, 4 in the eyeballs, and 12 at variable distances.

As part of his study, Armstrong included a “Mr. A. G. C”, a gifted visualizer, who was not in the student sample. Armstrong states the following: “His images are so habitually localized as in reality” and “he is also endowed with the gift of mentally ‘seeing around objects’, and he could image four faces of a die without difficulty. The visualization of all six faces, however, costs him an effort of attention, and when he was asked to accomplish it, it was noted that the hand which he held in front of him to aid his visual imagination (as though the die were held in it) *moved up toward the eyes*. Questioned about the fact, Mr. C. recognized the movement, although he had performed it involuntarily, and was able to estimate it in inches since his business and habits accustom him to measurement. Without strain he holds the hand and visualizes images at about 14 inches from the eyes; with strain they move up to a distance of 4 inches”.

Commenting on his study, Armstrong noted that “the most striking phenomenon shown is the intimate relation of imagery and attention and the effect of the latter on the various phases and characteristics of the former”. He noted that women students from Wesleyan and Vassar College showed a “higher development of the faculty” of mental imagery than the sample of men ([27], p. 506). Armstrong’s findings from his sample of men fully replicated those of Galton and double the weight of the latter’s conclusions.

### 2.4. Fernald’s Studies

Mabel Ruth Fernald presented her PhD dissertation in 1912 at Princeton University, USA, on “The Diagnosis of Mental Imagery” [28]. With references to Fechner (1860), Galton (1880, 1883) and Armstrong (1894), Fernald’s dissertation contained no less than 118 references, which is indicative of the intense scholarly interest in mental imagery during this period. For her own introspective study, Fernald chose 11 participants who were instructors or advanced graduate students trained in introspection. The nature of that training was not specified. Five were men (A, Ad, Hs, P and S) and six were women (C, G, H, Sun, T and V). For the first time in a phenomenological study, the investigator and majority of participants were women. The tests were conducted at the University of Chicago during the period of 1908 to 1910.

The participants were given a variety of “diagnostic” tests, and the current review is confined to the results for reading. In contrast to Fechner, Galton and Armstrong, who studied voluntary imagery evoked by instructions or questionnaires, Fernald’s test procedures evoked imagery that occurred spontaneously while reading evocative passages. Twenty-one short passages were used with descriptive material in varying levels of abstractness–concreteness and referring to varied sensory fields. Fernald’s procedures included silent reading at a normal rate with the content reproduced, silent reading at faster rates with the content reproduced, silent reading at a normal rate without reproduction, the passage read out loud by the investigator and the passage read aloud by the participant. The participants reported their introspections on (a) the verbal imagery used; (b) the illustrative imagery most commonly used; and (c) a statement on the nature and amounts of these two imagery kinds. Table 3 shows the findings for each participant, including their overall imagery ability as rated by Fernald, and the incidence of imagery of six different kinds for each participant over the readings. The range of variation is moderately high, with the incidence of tactile, organic or visceral imagery reported by all 11 participants, and temperature (warmth, cold) by five. It is notable that eight of the eleven participants reported at least five of the six kinds of imagery, with one participant (H) rated as having “minimal” imagery ability, reporting only three of the six kinds.

Fernald’s findings enable the following conclusions:

(i) The range of individual differences was large. However, every one of the eleven participants reported spontaneous illustrative mental imagery while reading;
(ii) Even participants who reported scant or minimal amounts of illustrative imagery still experienced reading as illustrated by mental imagery of at least three kinds;
(iii) The high incidence of tactile, organic or visuo-motor imagery, which was reported by all participants, was striking;
(iv) Visuo-motor imagery was prevalent, with only one of the eleven participants reporting an absolute lack of visuo-motor illustrative imagery;
(v) In line with (iv), coloured imagery was present for ten of the eleven participants;
(vi) Auditory imagery was present in all but three cases (H, S and Sun) but, overall, less frequently than visual imagery;
(vii) The sample size was limited, and potential gender differences could not be statistically tested with the data from this study.

As a general comment, we should not be surprised that Fernald pointed to a difficulty of articulation for “even the best” of her introspectionists, whom “had been so absorbed in the operation that they could not tell how they had done it” ([28], p. 136). This question is raised repeatedly throughout phenomenological research and is impossible to extinguish because it is palpably valid. The method of introspection fell into disuse after 1913, when the American behaviourist John B. Watson proposed that psychology could manage without it and any other method for research on consciousness [29]. Watson advocated a psychology that he claimed was “a purely objective branch of natural science”, and behaviourist methods with animal experiments on learning and memory replaced introspection. As a consequence, almost no phenomenological studies occurred in psychology between 1930 and 1957, when a book by TPH (Peter) McKellar called *Imagination and Thinking* appeared [30]. We turn next to VMI studies concerned with eidetic imagery.

### 2.5. Early Studies of Eidetic R. Imagery

A starting point for studies of eidetic imagery is Erich R. Jaensch [31,32], working at Marburg, Germany, who defined eidetic imagery in 1923 thus:

*“Optical perceptual (or eidetic) images are phenomena that take up an intermediate position between sensations and images. Like ordinary physiological after-images, they are always seen in the literal sense. They have this property of necessity and under all conditions, and share it with sensations. In other respects they can also exhibit the properties of images (Vorstellungen). In those cases in which the imagination has little influence, they are merely modified afterimages, deviating from the norm in a definite way, and when that influence is nearly, or completely zero, we can look upon them as slightly intensified after-images. In the other limiting case, when the influence of the imagination is at its maximum, they are ideas that, like after-images, are projected outward and literally seen.”*

([31], pp. 1–2)

Jaensch’s statement continues:

*“For the great majority of adults there is an unbridgeable gulf between sensations and images. It has always been known that for a few individuals this is not true. Some people have peculiar ‘ intermediate experiences ’ between sensations and images. From the description that such people have given of these experiences, and from the characterization we have just given of eidetic images, we must conclude that their ‘ experiences ’ are due to eidetic images. These phenomena, it is true, are rare among average adults.”*

([31], pp. 3–4)

According to Jaensch [31], “a positive El is a strong form of visual after-image having great clarity and in the colours of the stimulus object, often in very fine detail”. In Jaensch’s view, “the frequency of eidetic imagery is highest in young children. Its opposite, the negative El, appears in complementary colours”.

Jaensch used the observed individual differences in eidetic imagery ability to develop a speculative typological system, which Jaensch opportunistically altered and aligned with the racist philosophy of national socialism in 1930s Germany. Attempts by independent psychologists to replicate Jaensch’s findings failed, and the resulting theoretical controversy led to Jaensch’s work becoming discredited, e.g., [33,34].

Allport (1924) [33] pointed out that eidetic images are definitely not photographic because of their “flexibility”, in which they frequently metamorphose with extensive, unconscious and uncontrolled changes, and have a frequent incompleteness of contents. It had also been reported that eidetic imagery may arise spontaneously with no specific evoking perceptual stimulus [31,32]. The Marburg School used the images’ spontaneous appearance as an indicator of the “B-type”. In the case of the “B-type”, according to Klüver [34], “the eidetic image is often nothing but a visualized idea projected into perceived space… He can, without any effort, produce eidetic images and, at pleasure, vanish them; he can do this without a preceding presentation of a stimulus…” (p. 181). In the next section, eidetic imagery phenomenology is discussed in more depth and detail.

## 3. The Middle Period, 1930–1999

The middle period began in what—from the perspective of phenomenological research—could be called “The Dark Ages”. For 50 years, from 1913 to 1963, phenomenological research on human mental life was effectively “cancelled” and replaced by experimental, behavioural studies of learning and memory. For mainstream psychological science, mental imagery and consciousness were “beyond the pale” and were deemed not to exist. Ultimately, mental imagery research was actively resumed in the 1960s, when detailed phenomenological and experimental studies together began to reveal a high degree of consistency across studies. This section begins with two contrasting approaches to eidetic imagery, one viewing it as “reproductive” and the other viewing it as “constructive”. The former approach gradually diminished towards a common approach based on the principle that mental images are phenomenological constructions.

### 3.1. The Myth of Eidetic Imagery as “Photographic” Memory

One hypothesis that originated with Jaensch and received continued attention was the idea that eidetic imagery is a so-called “photographic” memory, in which any visual stimulus—allegedly—can be held as a lasting, accurate, projected image. For example, Gengerelli [35] observed a young woman who could correctly discriminate tiny differences in the diameters of mentally imaged circles compared to a standard stimulus presented at some distance. The majority of studies on eidetic imagery have used the Easel Test. A blank piece of grey card, 24 by 30 inches, is placed on a support. First, the participant is approximately 20 inches in front of the screen, fixating on a red, four-by-four-inch square for 10 s, then, after removal of the stimulus, reports what they subsequently “see” on the screen. Successively, the procedure is repeated with blue, black and yellow square stimuli. Being used to seeing images—most people report negative after-images—the participant is shown a silhouette drawing, purely black-and-white, which they scan for one half-minute then, when it is removed, indicate what they image on the screen. If they report an eidetic imagery, they are questioned about its content; otherwise, they are asked about the picture which they had just previously viewed. Three more pictures are presented in a sequence: one more silhouette and two full-colour pictures. The four pictures originally were chosen for North American children: silhouettes showing a family scene and an Indigenous American hunting (both containing animals); the coloured ones feature another Indigenous American fishing and a scene from “Alice in Wonderland” showing Alice with the Cheshire cat

Based on studies with children of varying ages, the hypothesis of “photographic” memory has been investigated with generally inconsistent findings. Ralph N. Haber [36,37] used the Easel Test with five behavioural criteria for differentiating eidetic imagery from afterimagery. Eidetic imagery occurred when (i) saccades occur during the VMI of the stimulus, with the image remaining still; (ii) the image has a protracted duration, and appears in the original colours; (iii) the eidetic image is projected into space to fall on any available surface; (iv) the present tense is used by the participant when reporting images, not the past tense; (v) two images can be superimposed and reported as a composite [37]. During a five-year period, however, Haber struggled to find only 20 children (less than one per cent of his sample) with eidetic imagery as the so-called “photographic” type of memory, which forced the conclusion that *“eidetic images are only available to a small percentage of children 6–12 years old, and are virtually nonexistent in adults. However, extensive research has failed to demonstrate consistent correlates between the presence of eidetic imagery and any cognitive, intellectual, neurological, or emotional measure.”* [37].

Haber devised a more direct test by showing two pictures in sequence, which, when superimposed, formed a third image showing a person’s face. Four participants reported seeing the facial eidetic imagery, but this test was insufficiently stringent because the children might have guessed the composite face from either picture alone. Then, a Harvard student claimed she could “hallucinate at will a beard on a clean-shaven man or repeat a page of poetry written in a foreign language verbatim—backwards and forwards—after simply looking at it” [38]. Stromeyer and Psotka [39] attempted to demonstrate this person’s ability using a new technique involving random dot stereograms. However, demand characteristics may have fallen into play and one investigator reported a photographic eidetic imagery memory incidence of “none in a million” [40] in the general population. The possible reasons for this failure will be indicated in the following sections.

### 3.2. Cross-Cultural Study with Participants from Five African Countries

Leonard W. Doob (1966) [41] conducted cross-cultural studies of eidetic imagery in five African countries, using Haber’s methods with the Easel Test and culturally adapted materials. Doob found it difficult to consistently evoke after-images, and so he extended the exposure time to 15 s. Because Haber’s pictures were “culturally saturated”, Doob substituted photographs taken in local African settings such as a picture of African women with infants and metal rooves (sic) on their heads and a scene in colour of a bus with a full load of passengers. Additionally, complicated montages were used to test the accuracy of the detail of eidetic imagery or picture memory, e.g., a white elephant beside a white tree, with a multi-coloured car above, and a yellow giraffe and green elephant beside a yellow-coloured tree below. The examiner sat opposite the participant at a small table so they could see how well the instructions to keep the eyes fixed on the colours and to scan the pictures were obeyed, and whether the participant’s attention was directed to the screen during the reporting of eidetic imagery. Virtually all testing occurred out-of-doors in varying, poorly controlled conditions. The investigator notes that it was field research requiring a standardization of procedures that would often have fallen short of achieving the methodological ideal.

Samples of adults were tested in five African societies: a group of Ibo in Eastern Nigeria (N = 28; 39%); a sample of Kamba in Central Kenya (N = 49; 39%); a nomadic group of Somali (N = 24; 4%); a nomadic group of Masai in Kenya (N = 20; 80%); and a mixed Swahili-speaking group in eastern Tanzania (N = 33; 15%). The percentages show the proportion of each sample reporting some eidetic ability. The following quotations represent four of the positive responders’ experiences:

*lBO CULTIVATOR: I see human beings. (How many are there, can you count them?) Yes, four [five].*

*KAMBA WOMAN: I see some people and motor cars, one man on top, with goods on top. (What is the color of the bus?) Looks like red and green [brownish and white]. (What else do you see?) Many people.(Do you see any trees?) Yes. (Where?) [Points correctly]. (Do you see anything else?) I see nothing more. (Can you see the license plates on the cars?) No.*

*MASAI HERDER: (Can you see their trousers?) No, I can’t see them. (What do you see on the screen now?) I still can see that person, but it is not now very clear.*

*SWAHILI CULTIVATOR: I see some numbers, an elephant, a man. (Do you see them clearly?) I see them. (Can you see the numbers?) I see the numbers clearly. (Can you read them?) I can read them. (Would you read them, please? Tell us what numbers you see on the screen now.) 0 [pause of 7 s] 2 [5 s] 2 [4 s] 1 [5 s] 7 [6 s] 0 again [actually: 0714653282760]*.

([41], pp. 22–23)

Doob reported: “Almost without exception those with eidetic imagery…stated that they had been amazed to see the images on the screen, that this was an experience they had never or seldom previously had (Masai 7/9; Swahili 7/8; Kamba 22/25)”. However, this latter finding was most likely a consequence of the participants’ unfamiliarity with being tested by looking at pictures on easels because the majority claimed they customarily were used to “seeing pictures in front of my eyes” (projected imagery) (Somali 2/6; Masai 15/21; Swahili 18/32; Kamba children 45/57). There was no statistically significant relationship between the presence of eidetic imagery and of projected imagery in any of the groups. Likewise, many informants stated that often they have “pictures in my head,” or pictorial images when recalling events or people from the past (Somali 6/9; Masai 13/14; Swahili 6/16; Kamba 66/86). Pictorial images were occasionally reported by Somalis to be “in my heart” and by the Swahili to be “in my mind”. No association existed between eidetic imagery and pictorial imagery, nor between eidetic imagery and projected imagery. During or after the tests for eidetic imagery, the accuracy of recall was also found to be unrelated to claims of projected imagery or pictorial imagery for the pictures that had been exposed. A Somali stated that he could see “the bus” in his head but, when asked to describe what he saw on top of the bus, he incorrectly replied that “nothing” was there. From the standpoint of most informants questioned, there was a difference between remembering with eidetic, projected or pictorial imagery on the one hand, and what is ordinarily called “simple memory” or recall on the other (Somali 15/15; Masai 4/4; Swahili 7/8; Kamba adults 14/14). Doob’s [41] observations lead to the following conclusions:

(i) The incidence of eidetic imagery is not significantly higher or lower in African societies than in European or US samples;
(ii) A high degree of variability was evident within each sample and “the attributes and characteristics of eidetic imagery when they do appear do not vary from society to society and, in fact, are no different from those found in Europe and America” ([41], p. 29);
(iii) As was the case in the Western laboratory studies, the differences within groups appeared much greater than those between groups.

### 3.3. A Rekindling of Phenomenological Research on VMI

With the exception of the above-mentioned studies on eidetic imagery, the absence of any new phenomenological studies for almost half a century left a vacuum that remained unfilled until the 1960s and 1970s, when an “explosion” of mental imagery studies occurred. The 1957 book by Peter McKellar, *Imagination and Thinking* [40], presaged a re-awakening of psychological interest in mental imagery. Between 1957 and 1975, a productive group of researchers made substantial contributions to our understanding of individual differences in VMI. In 1974, a Japanese psychologist Takeo Hatakeyama [42,43] published a ground-breaking study of eidetic imagery in a 20-year-old woman (Y.K.). This fascinating case appeared as a pinnacle of eidetic imagery at the high ability end of the distribution. Hatakeyama’s research papers are available in Tohoku University’s repository at: https://cir.nii.ac.jp/all?q=Tohoku%20psychologica%20folia. Until now, they have not been widely disseminated and they warrant closer attention. The original purpose was to determine, through the use of VMI, whether Y.K. could fill a empty circle on a small, neutral card spontaneously with mentally imaged colour. The author used what later became known as the “Open Circle Test”, which is described as follows:

*One of the twenty subjects, YK, could see inside of the circle vivid images of concrete shape and colour, not mere colour image, at any time when a certain colour name was given to her. She gave a signal by tapping the desk with her right-hand forefinger, every time such an eidetic image appeared or it changed in its figure. She could also scan the images as if she were looking at paintings or photographs*.

[42]

The investigator used a white card (12.5 cm by 20 cm) and a grey card (10 cm by 15 cm). A circle of 5 cm in diameter was drawn in the centre of the white card, and one of 2 cm on the grey one. The investigator asked the participant to imagine a blue colour inside the circle, and then red, yellow and green. With the white card first and then the grey card, 3 min were assigned to each of the four colours (eight tests in total). After each occasion, Y.K. was required to state whether a colour image appeared, and if it did, what it looked like. They were requested to draw an image if they saw one, on a sheet of paper with a pencil.

**Figure 3:**
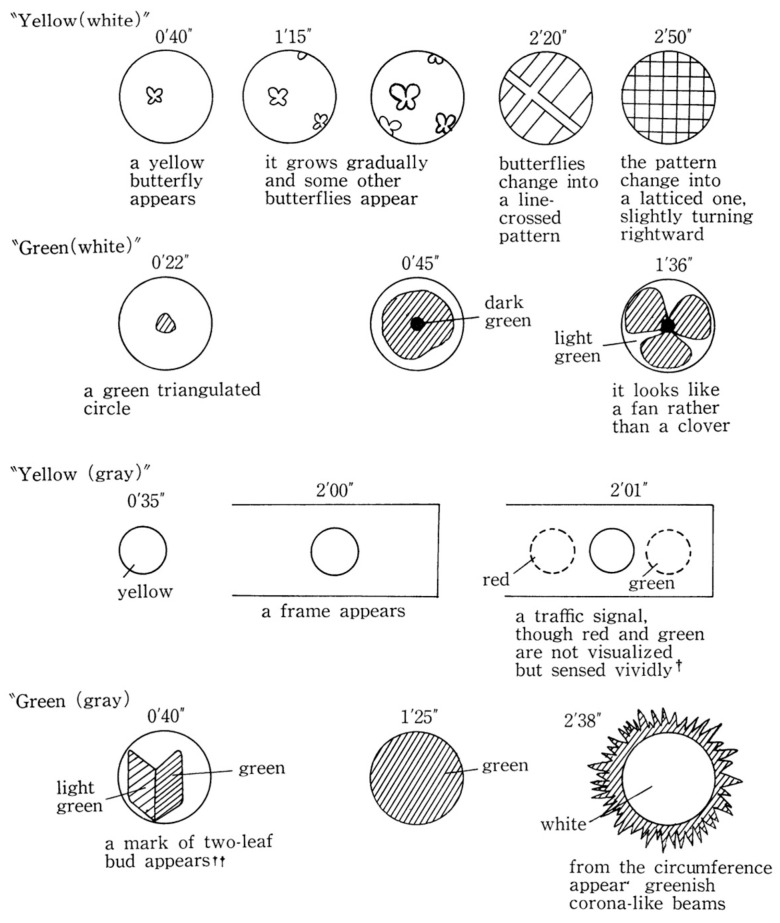
Y.K.’s original drawings in response to the Open Circle Test, with colouration as indicated [32]. “Blue,” “Red,” “Yellow,” and “Green” correspond to the appointed colours; “white” and “grey” to the cards; numerals to the passage of time within the assigned 3 min. “†: At this time of the test, Y.K. was going to a drivers’ school every day to get a license. However, the positions of “red” and “green” lamps in the imagined signal are contrary to the originals. ††: This mark must be placed on an automobile by beginner drivers in Japan. In the image, the yellow leaf of the original mark (the left-hand leaf) is altered to appear greenish. The drawings were produced by Y.K. The colours correspond to the participant’s descriptions and are indicative only.” Reproduced from [42].

Two months after the first laboratory visit, Y.K. came to two more sessions using the same methods. Twenty-four tests were carried out. The findings showed that, at each test, Y.K. could project images associated with the appointed colour names; the VMI content changed over the 3 min of the test. Y.K. _tapped_ the desk with her forefinger to indicate that an image had begun to appear inside the circle or if it transformed its shape. Y.K. produced drawings afterwards, and her responses to nine of the twenty-four tests are reproduced in Figure 3.

Hatakeyama’s conclusion was that eidetic imagery is a constructive process similar to those of the other VMI kinds of memory and imagination imagery. Hatakeyama (1975) [43] stated the following:

*Various experimental evidences given by a twenty-year-old female of extremely high eidetic ability shows that eidetic imagery is possessed of the constructive character. The word “constructive” means not only the fact that different features from the original stimulus appear in the image, but also, more positively speaking, that eidetic imagery has an aspect of being constructed like any other ordinary memory imagery. In this regard, it deserves emphasis that an eidetic image belongs to “imagery” and that it should not be construed merely as a photographic image, that is, an accurate copy of the original stimulus, or as a photographic memory, that is, an accurate retention of the original stimulus in visual memory.*

Thus, eidetic imagery appears to be a highly flexible and fluid form of VMI that does not necessarily require an evoking stimulus. Hatakeyama [42,43] divided his observations into five classes.

#### 3.3.1. Spontaneous Eidetic Imagery Not Based on a Stimulus Presentation

As noted, Y.K. visualized vivid eidetic images inside the circle at any time colour names were given to her. There were some cases when the content of a produced image could not be identified, or when part of the image was discrepant with the original object that had been indicated (Figure 3). When Y.K. was asked to visualize “a small concrete object” on a projection screen (50 cm by 40 cm, placed 50 cm in front of her eyes), the image showed alterations in certain parts. Details of the objects were found to be incorrect, e.g., the image of a compact, the inside mirror, which could not be seen from the outside, was visualized, but the shape of the mirror was round, whereas her own compact had a square mirror.

#### 3.3.2. Eidetic Imagery Elicited from Stimulus Presentation

Observations in [43] show highly constructive, sometimes inaccurate, characteristics of eidetic imagery, which included the following:

(i) Gradual Development and Fading;
(ii) Attentional Effort;
(iii) Supplementation: “new features, which the stimulus pictures did not contain, were added to the images”;
(iv) Grounding: “where images lacked a particular part, such part was not left entirely blank but suffused as some kind of ground in the image”;
(v) Metamorphosis: dramatic and sudden changes in eidetic imagery content;
(vi) Re-emergence;
(vii) Replication: e.g., “an orange square was presented and the same colour image appeared”;
(viii) Figure–Ground Colour Reversal.

#### 3.3.3. Eidetic Imagery Reproduced after a Lapse of Time

Eidetic images reproduced after a lapse of time revealed an “exceedingly constructive character”. It took time before the eidetic images were produced. These images tended to appear “little by little”. Details of the image were often initially indistinct and became more distinct. There were instances where motion or activity appeared in the image, e.g., “in the image of one silhouette picture evoked on the following day, a flag on the top of the building in the original picture, was seen fluttering”. When the picture of the “Sparrows’ School” was evoked on the following day, “the sparrows were moving about inside the nest, bobbing their heads; at the same time S sensed them peeping”. There were some cases of divergence between the eidetic image and the memory contents of the original stimulus picture.

#### 3.3.4. Eidetic Imagery Projected on the Surfaces with Complicated Patterns

When the projection cards presented complex designs, such as patterns of chiyogami (rice-paper with coloured figures) or a black-and-white striped pattern, eidetic images were constructed to assimilate elements of the projection surface.

#### 3.3.5. Eidetic Imagery Evoked from Complex and Abstract Stimulus Figures

Tests with complex, abstract stimuli of Chinese characters used complicated numeral patterns (e.g., “twenty numerals drawn in blue, red, yellow or green, and scattered in various directions”). The eidetic imagery here were far from complete reproductions of the original stimuli, with “extremely constructive features”, which lacked unity with other parts. The “entire image did not appear at the same time, but a restricted part, to which YK directed her eyes on the projection surface, was visualized and, as she shifted her eyes, the missing part appeared”.

Hatakeyama’s research [42,43] led to some interestingly novel conclusions:

(i) EI is a constructive process of agency involving the participant’s attentional efforts, force of will and intentions. This point is reminiscent of Fechner’s and Galton’s findings;
(ii) EI is a flexible and fluid process with gradual development and fading, attentional effort, supplementation, grounding, metamorphosis and figure–ground colour reversal;
(iii) As suggested by Fechner, there is a dynamic interaction between the after-image and the eidetic image.

This study with a young woman of high eidetic imagery ability indicates the pinnacle of eidetic imagery achievement. Further studies by Hatakeyama [43] in different student age groups replicated Fechner’s earlier-reported finding that after-images, following Emmert’s Law, could be induced with eidetic imagery in one or more 20–24-year-olds, and more easily in children. To gain a realistic impression of eidetic imagery ability in the normal range, a study with a representative sample of the adult general population was necessary, as described next.

### 3.4. Replication of Hatakeyama’s Findings

**Figure 4:**
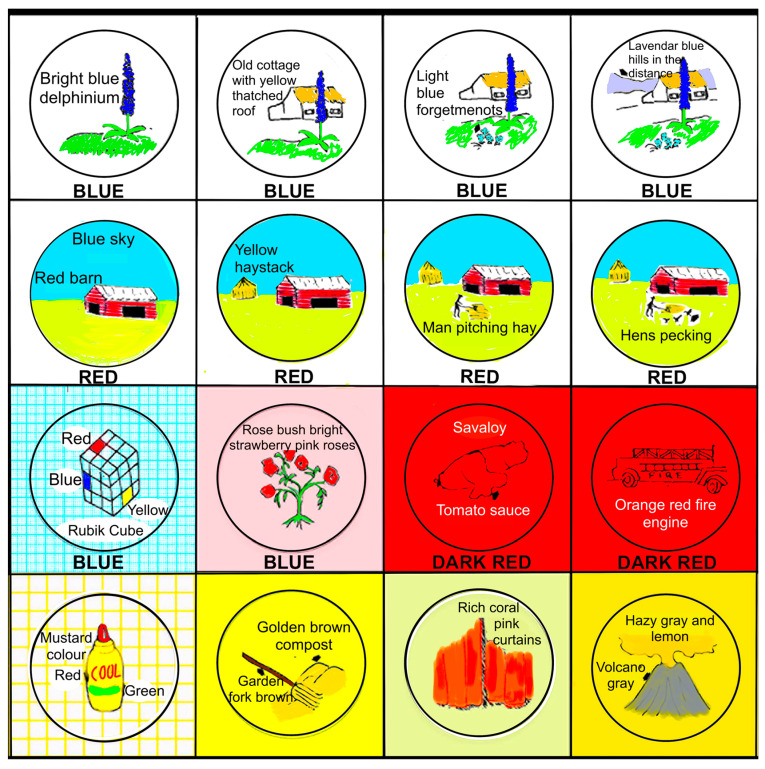
A high-ability participant’s eidetic responses to ten trials with the Open Circle Test. Original black and white drawings were produced by the participant, with colouration as indicated. The top two rows show images evoked in each of two trials with a single colour name (trial 1:blue, and trial 2: red) over a five-minute period. The bottom two rows show responses for each of eight trials with varying papers and colours. The paper colour is shown (top two rows, white) and the evoking stimulus colour name, which was presented aurally, is printed beneath each circle. For the two tests using the dark red paper, the paper was stippled (stippling unshown) and elements of the produced images were assimilated with the stippling (replicating Hatakeyama’s finding, Section 3.3.4). For the four trials in the bottom row, no colour name was presented, and the responses were evoked simply by the participant looking at the circle printed on four differently coloured papers. Notice how the four papers of varying shades of yellow spontaneously produced a diverse set of VMI [44].

Two replication studies of Hatakeyama’s findings were conducted by the author and Peter McKellar at the University of Otago, New Zealand [44]. Initially, we tested two eidetikers at the high end of the ability range. Our data replicated, in every detail, Hatakeyama’s findings with Y.K. [42,43] (Figure 4). All of the characteristics previously observed by Hatakeyama [42,43] were repeated: gradual development and fading, attentional effort, supplementation, assimilation, grounding, fragmentation, metamorphosis and figure–ground colour reversal. 

Next, we presented the Open Circle Test to randomly selected samples from the student population. Thirty of forty-four (68.2%) unselected students gave positive eidetic imagery reports in response to specific colour names [44]. Using less stringent criteria for eidetic imagery (e.g., the ability to see VMI projected in clouds or wallpaper patterns), eidetic imagery was reported by 100% of the sample. A strong relationship was obtained between the two types of eidetic imagery, “typographic” vs. “spontaneous” eidetic imagery, using the Easel Test and the Open Circle Test, respectively, in which all participants who could produce the former also produced the latter. Another replication by [45] with 327 students also showed a significant relationship between “typographic” eidetic imagery in the Easel Test and “voluntary or spontaneous” eidetic imagery, suggesting that these two kinds of eidetic imagery are essentially on a continuum of common ability.

Studies of eidetic imagery using unselected student samples suggest these conclusions:

(i) Eidetic imagery is a vivid, fluid and flexible process;
(ii) Eidetic imagery is a constructive process, not a reproductive or “photographic” one;
(iii) Eidetic imagery can be voluntarily controlled by the participant’s focus of attention;
(iv) Like all other kinds of VMI, there are wide individual differences;
(v) In its weakest, spontaneous form, eidetic imagery is available to the entire population.

## 4. Recent Period (2000–2023)

### Mental Imagery in People with Intellectual Disabilities

There are only a few studies of mental imagery in people with intellectual disabilities. In the recent period, there have been efforts to fill this gap. Brown and Bullitis [46] explored mental imagery in 16 adults with intellectual disability (ID) in comparison to a group of 10 students who were seen individually in a “bare, windowless room”. Photographs were briefly presented showing the following objects: (1) a tent, (2) a fire in a hearth, (3) a garden with a pool, (4) a woman doing a limbo exercise, (5) a man blowing out candles on a cake, (6) a woman by a step-ladder with a cup in her hand, (7) a chef cutting food, (8) a boat on a lake and (9) hikers in the mountains. Following the removal of the material, each individual was asked to describe any mental imagery that was evoked by the pictures. All respondents were video-taped, and their verbal responses were analysed. The ability to manipulate imagery was found to differ significantly between individuals and groups. However, all participants in both groups described a visual image spontaneously or when “provoked”, and colour was spontaneously described by 15/16 ID individuals and by only 4/10 controls. A majority of both groups reported multi-sensory imagery and displayed signs of emotion and of movement.

**Figure 5:**
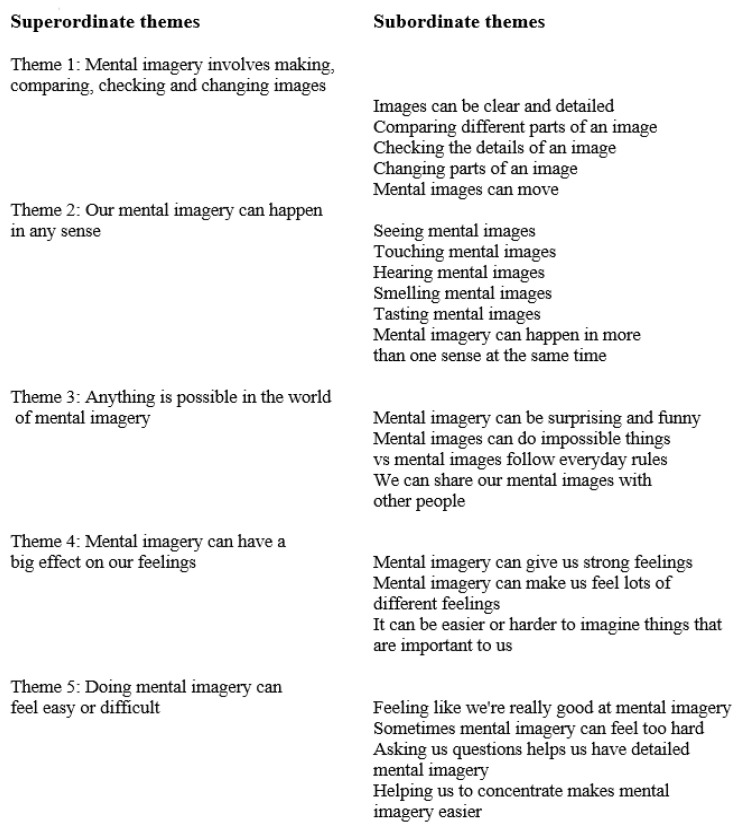
Themes and subthemes identified by Hewitt et al. ([47], Creative Commons).

Another new dataset has been provided by Hewitt et al. [47], who used participant-created drawings and semi-structured interviews to study the phenomenology of mental imagery in an ID group. The participants were ten people, two men and eight women aged from 25 to 54 (mean age = 52.3 years), who were assessed as having mild-to-moderate intellectual disabilities. They were invited to create an image, by drawing and/or by making a collage, to represent their experience of mental imagery. Participants were guided by the investigator to give a description of the image in its different parts and describe its overall meaning and the process of creating it. The *Vividness of Visual Imagery Questionnaire* [48] was presented together with prompts to explore the vividness levels of their mental imagery. The interviews took between 48 and 93 min (M = 62 min). The transcripts and images were analysed using interpretative phenomenological analysis [49]. The results are shown in Figure 5.

Hewitt et al.’s study suggests the following conclusions:

(i) People with ID experience and engage with mental imagery across different sensory modalities;
(ii) The clarity, detail and richness of the images is notable. As well as creating mental images, participants described changing and manipulating these images with VMI metamorphosis as observed by previous investigators described above;
(iii) Images could be “surprising, extraordinary and humorous”;
(iv) Almost all participants described feelings associated with mental imagery, which “could be strong and powerful and encompassed a wide range of different emotions, such as surprise, delight, disgust and fear”;
(v) There is a continuum of “mastery over mental imagery”, with wide individual differences, as observed in all of the VMI studies reviewed here.

Another recent study [50] found no significant differences in VMI vividness or eidetic imagery ability in people with intellectual disability compared to typically developing individuals.

## 5. Synthesis of Findings

This review summarizes the findings of a specialized array of phenomenological investigations of VMI covering the entire history of mental imagery research. The review has collated, into a single analysis, a unique database that is drawn from a rich diversity of participants, beginning with Gustav Fechner (1860); then Galton’s (1880) 100 distinguished “men of science’; samples of US university students; German, Japanese and New Zealand eidetic imagers; Africans in five countries on that continent; and groups of contemporary Britons with intellectual disabilities. In each and every group, there existed a widespread variability in the nature and vividness of VMI, but there is no obvious distinction in overall VMI abilities and characteristics between the different participant groups. The reviewed findings indicate an extraordinarily high level of consistency, replicability and coherence in phenomena that were written off for half a century by the behavioural school of psychology as “unscientific”.

**Figure 6:**
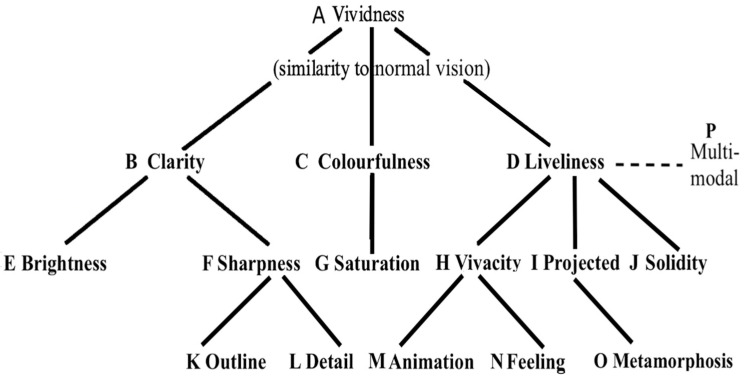
Taxonomy of VMI vividness based on the reviewed phenomenological studies. This hierarchical structure has four levels, with each term falling above and below its subordinate and superordinate constructs, respectively. The letters A–P indicate 16 interrelated characteristics that are tabulated across studies in Table 4.

The purpose remains to synthesize the present findings in the form of a template that specifies the properties of VMI in healthy adult humans. The findings have comprised quantitative and qualitative data of a verbal and graphic nature on VMI phenomenology. This synthesis is inductive in nature, while taking into account the frequency of each of a complex of 16 characteristics across the ten datasets. The entire corpus of findings is synthesized in Figure 6. The letters “A, B, C, D…” label the 16 features to enable cross-referencing between Figure 6 and Table 4.

It can be seen that the template of VMI characteristics ranges from the most general at the top of the hierarchy (“Vividness”) to the most specific at the bottom of the hierarchy (“Outline”, “Detail”, “Animation”, “Feeling”, etc.). To guide the construction of the template, it was necessary to weigh the relative importance of any descriptive term employed in the VMI phenomenological accounts using the frequency of each term as a proxy measure of a term’s relevance and prominence.

The most frequently mentioned attribute of vivid VMI in the research participants’ descriptions across the ten reviewed studies has been “colour”, and the term “Colourfulness” is therefore included in the second tier of the template hierarchy, together with “Clarity” and “Liveliness”.

The taxonomic rank at each level has been applied to the phenomenological descriptors of VMI vividness, according to their frequencies in the datasets in combination with their conceptual similarities and differences. The taxonomy started by establishing the taxonomic ranks in broad categories like “Low Vividness,” “Moderate Vividness,” and “High Vividness”, and then proceeded with defining a second tier of specificity: “Clarity”, “Colourfulness” and “Liveliness”, and then a third and a fourth tier in a hierarchy.

Next, the content of the data from the reviewed studies suggested many other characteristics for classification that distinguish one level of vividness from another, including “Brightness”, “Sharpness”, “Saturation”, “Vivacity”, “Projected” and “Solidity”. As noted, the categories are arranged hierarchically, with the most general one at the top (“Vividness”) and more specific subcategories below (i.e., “Clarity”, “Colourfulness”, “Liveliness”). The fourth row indicates the most specific level of VMI characteristics: “Outline”, “Detail”, “Animation”, “Feeling” and “Metamorphosis”.

Table 4 indicates the extraordinarily high degree of consistency of findings across studies. A check mark in a cell indicates that the row study reported the column characteristic. A total of 155/160 cells are checked meaning that one indicator (N, Feeling) was unreported in three of the studies, and a second indicator (O, Metamorphosis) was unreported in two of the studies. These investigators did not solicit the necessary information about participants’ emotional feelings or metamorphosis, and these five cells remain blank.

## 6. Discussion

The present review examines, collates and synthesizes historical and contemporary datasets on the phenomenology of VMI. As suggested in the Introduction, the process of discovering the nature of VMI is reminiscent of the parable of the blind monks inspecting the different part of an elephant (Figure 1). The current research findings are unique for their level of detail, scope, breadth and depth of the reviewed literature. The end product is a template defining VMI vividness with 16 descriptors.

Until now, our understanding of VMI has been fragmentary and piecemeal. The VMI template is a step towards describing the VMI “elephant” in its entirety for the first time. In light of this synthesis, it is possible to provide as comprehensive a specification of VMI vividness as possible using extant data. Previous definitions of VMI vividness had included clarity and liveliness, but not colourfulness [51,52]. However, the prominence of terms referring to “colour” in every one of the ten reviewed studies require the inclusion of colourfulness as an essential component of VMI vividness. Notably, the principal instrument used for the assessment of vividness, *The Vividness of Visual Imagery Questionnaire* (VVIQ), refers to colour in eight of its sixteen items [53].

The historical knowledge vacuum left by the neglect of VMI phenomenology led to a variety of incomplete or erroneous theoretical speculations and inadequate clinical applications. To give an example, the “Imagery Debate” [8,9] embraced two adversarial positions about the nature of VMI that were boldly expressed yet both were notably inconsistent with VMI phenomenology revealed by the present synthesis of findings. The first position {8} argued that VMI involves an analogue representation that is viewed by the imager as a “picture in the mind” or “mental photograph”. The second position [9] argued that VMI involves tacit knowledge of the imaged situation, which causes the performance to proceed as the imager believes it should proceed if carried out perceptually. Neither the picture theory nor the tacit knowledge theory is consistent with the present findings. VMI *consists of a highly constructive and dynamic process that is both enactive and affective, having its own momentum, and often leading to unpredictable outcomes that can include the metamorphosis of the initial image content*. Thus, the present findings on VMI phenomenology are consistent with an enactive approach to perception and mental imagery, such as one proposed by this author [15,52].

Another example of a mismatch between assumptions and practice occurs in research attempting to link VMI content with the clinical condition of depression. Emily A. Holmes et al. [53] suggest that “individuals with depression may have particular difficulties with generating vivid and compelling imagery of positive events. Negative memories and images, in contrast, may come to mind all too readily” [53], i.e., depressed people experience depressing mental imagery. It is possible to escape the obvious tautology of this theory by exploring the links between VMI attributes such as colourfulness and depressive tendencies. For example, evoking colour imagery to determine whether people have a depressive tendency, the three colours of yellowish red, purple and dark grey are found to be significant discriminant variables [54]. These findings are consistent with the observation that Instagram photos posted by depressed individuals tend to be bluer, darker and greyer [55]. Using the VMI template in Figure 6, it is possible to make other predictions about the possible links between VMI and depressive mental states. For example, if the VMI of depressive people is explored over a period of minutes in the Open Circle Test, it can be predicted that they will observe image metamorphosis towards blue, dark and grey colours, towards less animation and lower detail compared to non-depressive people.

The template of Figure 6 can also be applied in future analyses of mental image vividness, which could lead to an improved understanding of how the 16 different vividness attributes contribute to the phenomenology of mental imagery. The current definition of “aphantasia”, the alleged congenital absence of voluntary VMI defined by low scores on the VVIQ [56], could be more accurately explored by using all 16 of the template characteristics rather than simply VVIQ overall vividness total scores alone. It appears likely that significant numbers of people currently “diagnosed” as “aphantasic” would show positive responses to the Open Circle Test, with all 16 of the characteristics that are available for the assessment. This form of testing appears likely to reveal that many (or all) so-called “diagnosed aphantasics” are misdiagnosed false positives due to the inadequate method used for “diagnosis” [57].

By applying the taxonomic rank to the 16 criteria of phenomenological experiences of mental image vividness, future investigators will be able to create a structured framework that enhances our understanding of mental imagery experiences and contributes to the broader fields of cognitive science, vision science and psychology more generally.

## 7. Strengths and Limitations

The main strengths of this review are: (i) that it integrates for the first time findings from a set of nine largely neglected studies together with a widely-cited, but misinterpreted, study by Galton; (ii) that the selected studies represent highly diverse samples from five continents and 163 years of controlled investigation; and (iii) the impressive degree of consistency between these ten sets of findings. The main limitations are: (i) the relative lack of studies using non-verbal measures of VMI and (ii) the omission of publications in languages other than English, German and French.

## 8. Conclusions

This review and synthesis of diverse VMI phenomenological studies in healthy adults serves as a unique resource for investigators of individual differences, cognitive development and clinical and neurological conditions. The review has revealed an extraordinary degree of consistency and replicability of VMI characteristics across time, space and culture. VMI is a constructive process, with a continuum of intensity or vividness as its most prominent and frequently reported feature. The discovered template specifies the properties of VMI in healthy adult humans, taking into account the weight of evidence drawn from ten primary studies. Vividness is defined as a combination of clarity, colourfulness and liveliness, where clarity consists of brightness and sharpness, colourfulness consists of saturation, and liveliness consists of vivacity, animation, feeling, solidity, projection in three-dimensional space and metamorphosis. 

The template provides a schema for further research on the phenomenological properties of VMI. The taxonomic template for the phenomenological experience of mental image vividness enhances our understanding of VMI experience and contributes to the broader fields of cognitive science, vision science and psychology more generally.

## Figures and Tables

**Table 1:** Showing the qualities of memory imagery reported by Fechner and six interviewees [21].

Identity	Age	Rank *	Easier with Eyes Open or Closed **	Vividness to Some Degree	Colour to Some Degree	Feelings of Pressure or Contraction or Effort ***	Project to An External Location	3 D	Multi- Sensory
**G Fechner**	59	=6	Open	Dim	Fleeting	+	+	+	+
**H Weisse**	59	=6	Open	Faint	Little	?	+	?	?
**A W Volkmann**	59	5	=	Faint	Faint	+	+	?	?
**W Hankel**	46	4	Open	Yes	+	+	+	?	?
**M W Robisch**	50s	3	=	High	+	+	+	+	+
**C M Fechner**	51	2	Closed	High	+	+	+	+	+
**M Busch**	39	1	?	Very high	+	+	+	+	+
**Total**	-	-	-	7/7	7/7	6/6	7/7	4/4	4/4

* Fechner’s ranking of each observer’s imagery strength. ** The participant’s own judgment about whether their visual imagery was stronger with eyes open or closed or of equal (=) strength.*** Feelings of pressure or tension in or around the eyes and/or head. + indicates the participant reported the characteristic in the column heading. ? indicates the datum was missing.

**Table 2:** Descriptive information on voluntary visual imagery by Galton’s three British samples, together with Armstrong’s US replication.

Sample	Complete or Partial Vividness	Colour Relatively Distinct and Natural	Extent of Field of View Larger Than, or Same as, Normal	External Projection Corresponded to Reality, or in Front of Eyes, at Least on Some Occasions
**Galton’s 100 men of science**	97/100	97/100	75/100	Missing data
**Galton’s sample of schoolboys**	167/172 ^	167/172 ^	47/121 *	126/160 **
**Sub-totals**	264/272 97.0%	264/272 97.0%	122/221 55.2%	126/160 78.8%
**Armstrong’s US** **students**	183/188 97.3%	183/188 97.3%	95/188 50.5%	153/188 81.4%
**Totals**	447/460 97.2%	447/460 97.2%	217/409 53.0%	279/348 80.2%

^ Estimate. * In 51 missing cases, the description was “insufficient”. ** Twelve missing data.

**Table 3:** Illustrative imagery while reading as reported by Fernald’s participants [28].

ID	Gender	Individual’s Overall Imagery Ability, as Rated by the Investigator	Tactile, Organic or Visceral Imagery	Visuo-Motor Imagery	Colour	Olfactory	Auditory Imagery	Temperature	Totals
**A**	Man	Minimum of illustrative imagery	+	+	+	+	+	No	5/6
**Ad**	Man	Minimal	+	+	+	+	+	+	6/6
**C**	Woman	Profuse visual imagery. Other imagery moderate.	+	+	+	+	+	No	5/6
**G**	Woman	Moderate amount of illustrative imagery	+	+	+	+	+	No	5/6
**Hs**	Man	Abundant imagery	+	+	+	+	+	No	5/6
**H**	Woman	Minimal	+	No	+	+	No	No	3/6
**P**	Man	Fair amount, but vague and indefinite	+	+	+	+	+	No	5/6
**S**	Man	Profuse, vivid and detailed	+	+	+	No	No	+	4/6
**Sun**	Woman	Scanty	+	+	No	+	No	+	4/6
**T**	Woman	Scanty, lack of detail, not vivid	+	+	+	No—only sniffed	+	+	5/6
**V**	Woman	Profusion of non-visual imagery	+	+	+	+	+	+	6/6
**TOTALS**			11/11	10/11	10/11	9/11	8/11	5/11	53/66

+: Indicates a positive report of a characteristic by a participant.

**Table 4:** Synthesis of the findings of ten primary studies of VMI phenomenology. Columns A–O correspond to the 16 VMI features in the template of Figure 6.

Study	A	B	C	D	E	F	G	H	I	J	K	L	M	N	O	P
**Fechner [21]**	✓	✓	✓	✓	✓	✓	✓	✓	✓	✓	✓	✓	✓	-	✓	✓
**Galton [24]**	✓	✓	✓	✓	✓	✓	✓	✓	✓	✓	✓	✓	✓	-	-	✓
**Armstrong [27]**	✓	✓	✓	✓	✓	✓	✓	✓	✓	✓	✓	✓	✓	-	-	✓
**Fernald [28]**	✓	✓	✓	✓	✓	✓	✓	✓	✓	✓	✓	✓	✓	✓	✓	✓
**Haber [36,37]**	✓	✓	✓	✓	✓	✓	✓	✓	✓	✓	✓	✓	✓	✓	✓	✓
**Doob [41]**	✓	✓	✓	✓	✓	✓	✓	✓	✓	✓	✓	✓	✓	✓	✓	✓
**Hatakeyama [42,43]**	✓	✓	✓	✓	✓	✓	✓	✓	✓	✓	✓	✓	✓	✓	✓	✓
**Marks and McKellar [44]**	✓	✓	✓	✓	✓	✓	✓	✓	✓	✓	✓	✓	✓	✓	✓	✓
**Brown and Bullitis [46]**	✓	✓	✓	✓	✓	✓	✓	✓	✓	✓	✓	✓	✓	✓	✓	✓
**Hewitt et al. [47]**	✓	✓	✓	✓	✓	✓	✓	✓	✓	✓	✓	✓	✓	✓	✓	✓
**TOTALS**	10	10	10	10	10	10	10	10	10	10	10	10	10	7	8	10

## Data Availability

Not applicable.
